# Dietary Supplemental Glutamine Enhances the Percentage of Circulating Endothelial Progenitor Cells in Mice with High-Fat Diet-Induced Obesity Subjected to Hind Limb Ischemia

**DOI:** 10.1155/2020/3153186

**Published:** 2020-02-13

**Authors:** Chi-Hsuan Ko, Sung-Ling Yeh, Chiu-Li Yeh

**Affiliations:** ^1^School of Nutrition and Health Sciences, College of Nutrition, Taipei Medical University, Taipei, Taiwan; ^2^Nutrition Research Center, Taipei Medical University Hospital, Taipei, Taiwan; ^3^Research Center of Geriatric Nutrition, College of Nutrition, Taipei Medical University, Taipei, Taiwan

## Abstract

This study investigated whether glutamine (GLN) pretreatment can enhance circulating endothelial progenitor cells (EPCs) and attenuate inflammatory reaction in high-fat diet-induced obese mice with limb ischemia. Mice were assigned to a normal control (NC), high-fat control (HC), limb ischemia (HI), and GLN limb ischemia (HG) groups. The NC group provided chow diet and treated as a negative control. Mice in the HC and HI groups were fed a high-fat diet which 60% energy provided by fat for 8 weeks. Mice in the HG group were fed the same diet for 4 weeks and then transferred to a high-fat diet with 25% of total protein nitrogen provided as GLN to replace part of the casein for the subsequent 4 weeks. After feeding 8 weeks, mice in the HC group were sham-operated, while the HI and HG groups underwent an operation to induce limb ischemia. All mice except the NC group were euthanized on either day 1 or 7 after the operation. The results showed that the 8 weeks' high-fat diet feeding resulted in obesity. The HG group had higher circulating EPCs on day 1 while muscle vascular endothelial growth factor, matrix metalloproteinase-9, and hypoxia-inducible factor-1 gene expressions were higher on day 7 postischemia than those of the HI group. The superoxide dismutase activity and reduced glutathione content in affected muscles were higher, whereas mRNA expressions of interleukin-6 and tumor necrosis factor-*α* were lower in the HG than those in the HI group. These findings suggest that obese mice pretreated with GLN-supplemented high-fat diet increased circulating EPC percentage, enhanced the antioxidant capacity, and attenuated inflammatory reactions in response to limb ischemia.

## 1. Introduction

Obesity, a complex multifaceted condition, has become an important worldwide health problem. Excessive accumulation of adipose tissues was shown to be associated with inflammation in metabolic tissues, called “meta-inflammation,” which may result in chronic low-grade inflammation and modest increases in circulating proinflammatory mediators [1]. Also, metainflammation is characterized by an impaired vascular structure and function in tissues and organs [2]. Obesity has been shown to be a predictor of cardiovascular diseases [3], and body mass index is positively associated with peripheral artery disease (PAD) [4]. Critical limb ischemia is a severe form of PAD. Lower-limb ischemia can cause claudication that eventually may lead to amputation [5]. Compared with normal weight, obese subjects demonstrated 3-5 times higher risk of PAD with limb ischemia [4].

Endothelial cells (ECs) line the blood vessels and form a barrier between the circulation and peripheral tissues. ECs regulate vascular function and are responsible for bidirectional communication between the endothelium and organs in which it resides [6]. Endothelial progenitor cells (EPCs) are progenitor cells which have the capacity to mobilize from bone marrow into the systemic circulation and subsequently differentiate into mature ECs to settle in impaired ischemic locations [7]. EPCs were proven to play important roles in reendothelialization and neoangiogenesis [7–9]. Previous studies revealed that compared to normal controls, the increased number of circulating EPCs in response to ischemia was remarkably decreased in obese mice [10–12]. Those findings implied that mobilization of EPCs from the bone marrow may be hindered in obese animals.

Glutamine (GLN) is an abundant amino acid in the body which plays important roles in modulating inflammatory responses, decreasing oxidative stress, and improving survival during critical conditions [13, 14]. GLN is an important energy substrate for rapidly proliferating cells [15]. It is considered critical for the differentiation, proliferation, and survival in various types of stem cells [16, 17]. Previous studies carried out by our laboratory showed that GLN supplementation promotes EPC mobilization in sepsis [18] and in diabetic mice with limb ischemia [19]. Although obesity also exerts inflammation and oxidative stress in the body, the metabolic alterations are distinct from sepsis and diabetes. Supplementation of GLN during obesity may exert different influences on EPC mobilization and vascular repair in a condition of limb ischemia. We hypothesized that GLN administration would attenuate inflammatory reaction and enhance EPC mobilization in obesity complicated with limb ischemia. In this study, we created a diet-induced obese mouse model to evaluate the influence of GLN on blood EPC changes and inflammatory reactions in response to limb ischemia.

## 2. Materials and Methods

### 2.1. Animals

5-week-old male C57BL/6 mice (weighing 20 g, *n* = 53) were used in this study. All mice were accommodated in standard conditions of temperature (21 ± 2°C) and relative humidity (50%~55%) with a 12 h light-dark cycle in the Laboratory Animal Center at Taipei Medical University (TMU). Feed and water were provided *ad libitum*. Care of laboratory animals was in compliance with the *Guide for the Care and Use of Laboratory Animals* (National Research Council, 1996). Experimental protocols were approved by TMU's Animal Care and Use Committee.

### 2.2. Experimental Procedure

Mice were randomly assigned to a normal control (NC, *n* = 5) group, a high-fat control (HC, *n* = 16) group, a high-fat limb ischemia (HI, *n* = 16) group, and a high-fat GLN limb ischemia (HG, *n* = 16) group. Mice in the NC group were fed a standard rodent chow diet (Purina no. 5001, Fort Worth, TX, USA) for 8 weeks, while mice in the HC and HI groups were provided a high-fat diet (60% kcal as fat) for 8 weeks [20]. Mice in the HG group were initially fed the same high-fat diet for 4 weeks, and then a high-fat diet supplemented with GLN was provided for the subsequent 4 weeks. The macronutrient distribution of the GLN-supplemented diet was identical to that of the high-fat diet except that part of the casein protein content was substituted with GLN which contained 25% nitrogen of the total amino acid (Table 1). This amount of GLN was used because it was proven to have an immunomodulatory property in various disease conditions [19, 21, 22]. At the end of the 8-week experimental period, limb ischemia surgery was performed in the HI and HG groups, and the HC group carried out a sham operation. Limb ischemia surgery was performed as previously described [23]. For anesthetization, mice were intraperitoneal injected with Rompun (10 mg/kg body weight (BW), Bayer, Leverkusen, Germany) and Zoletil (25 mg/kg BW, Virbac, Carros, France) which act as sedative and muscle relaxants, and then proximal and distal parts of the femoral artery were exposed and ligated. Buprenorphine (0.1 mg/kg BW) was injected subcutaneously every 12 h for 2 d of post operation to relief pain. After the sham or ischemic surgery, mice in the respective group were subdivided into two groups according to the sacrifice schedule on day 1 or 7 after surgery. All mice survived after the surgery. At the day of sacrifice, mice were anesthetized and euthanized by cardiac puncture. Blood specimens were collected in heparin-containing tubes. Plasma samples were separated from whole blood by centrifugation at 1500 × *g* and 4°C for 10 min. Epididymal tissues were weighed. The affected limb muscle (gastrocnemius) tissues were frozen in liquid nitrogen and stored at -80°C for further analysis.

### 2.3. Flow Cytometric Analysis of Circulating EPCs

Blood EPCs were measured according to the standard settings on a BD FACS CantoII flow cytometer (BD Biosciences, San Diego, CA, USA). Data were analyzed with BD FACSDiva™ v6.1.3 software (BD Biosciences). One hundred microliter fresh blood was incubated with fluorescein isothiocyanate- (FITC-) conjugated anti-mouse cluster of differentiation 34 (CD34; RAM34, eBioscience, San Diego, CA, USA), phycoerythrin- (PE-) conjugated anti-mouse CD133 (13A4, eBioscience), and allophycocyanin- (APC-) conjugated anti-mouse CD309 (Avas12a1, eBioscience). After incubation for 10 min at 4°C, lysing buffer (PharmLyse; BD Pharmingen, San Diego, CA, USA) was added to lyse the red blood cells and 2% paraformaldehyde was used to fix the cells. Fifty thousand cell populations were gated in the data analysis; cells show positive expression for CD34^+^/CD133^+^/CD309^+^ were considered as circulating EPCs.

### 2.4. Measurements of Plasma Concentrations of Adipokines

Leptin and adiponectin were analyzed by enzyme-linked immunosorbent assay (ELISA) kits (R&D Systems, Minneapolis, MN, USA) following the instructions of the manufacturer.

### 2.5. Measurement of Superoxide Dismutase (SOD) Activity and Reduced Glutathione (GSH) Contents in Muscle Tissues

Using a homogenizer, muscle tissues were prepared in a 250 mM sucrose solution containing 10 mM Hepes (pH 7.4) at 4°C to obtain 15% homogenates. The homogenates were centrifuged, and debris was discarded. Supernatants were used for the analysis of SOD activity (Randox, Antrim, Ireland) [24]. Total glutathione (tGSH) and oxidized glutathione (GSSG) were measured as previously described [21]. The GSH content was calculated as the difference between tGSH and GSSG. To analyze GSSG, 4 *μ*l of 10 mM 1-methyl-2-vinylpyridinium (Sigma, St. Louis, MO, USA) was added to 70 *μ*l of supernatants and remained at room temperature for 1 h. Then, buffer A (200 *μ*M NADPH, 2 unit/ml glutathione reductase, and 2 mM EDTA in 50 mM phosphate buffer) and buffer B (10 mM 5,5′-dithiobis-2-nitrobenzoic acid in 50 mM phosphate buffer) were added to 10 *μ*l of the samples immediately. The change in the optical density at 405 nm during 5 min was measured using a spectrophotometer. To measure tGSH, buffers A and B were added to red blood cells, and the absorption was recorded at 405 nm. The glutathione content was calculated according to the standard curve obtained from a known glutathione concentration. SOD and GSH were expressed based on the protein content. Protein levels of supernatants were measured by the Lowry method [25].

### 2.6. Messenger (m)RNA Extraction and a Real-Time Reverse-Transcription (RT) Quantitative Polymerase Chain Reaction (qPCR) Analysis

The TRIzol reagent (Invitrogen, Carlsbad, CA, USA) method was used to isolate total RNA from muscle tissue homogenates. The RNA pellets were dissolved in RNase-free water, and total RNA solution was stored at -80°C for the following assay. The determined and quantified of RNA was measured on a spectrophotometer at absorbances 260 and 280 nm. Synthesis of complementary (c)DNA from total RNA uses a RevertAid™ first-strand cDNA synthesis kit (Fermentas, Vilnius, Lithuania). cDNA was stored at -80°C for further analysis. The conversion of RNA to cDNA was performed by subsequent incubation for 5 min at 65°C, 60 min at 42°C, and 5 min at 70°C. Specific mRNA genes used in this study were amplified by a real-time RT-PCR using the 7300 Real-Time PCR System (Applied Biosystems, Foster City, CA, USA) with SYBR Green I. Primers used are presented in Table 2. Primers were determined based on deposited cDNA sequences (GenBank database, NCBI) and purchased from Mission Biotech (Taipei, Taiwan). Amplification of the gene was performed in 25 *μ*l containing 1x Power SYBR Green PCR Master Mix (Applied Biosystems), 400 nM for each primer, and 100 ng of cDNA. The reaction was processed by one cycle of 2 min at 50°C and 10 min at 95°C, followed by 40 cycles of 15 s at 95°C and 1 min at 60°C, with a final curve analysis. Levels of gene expression were quantified in duplicate by a real-time RT-PCR. Values of cycle threshold were normalized by mouse *β*-actin, and the relative quantity of mRNA expression was calculated.

### 2.7. Statistical Analysis

Data are presented as the means ± standard error of mean (SEM). GraphPad Prism 5 (GraphPad Software, La Jolla, CA, USA) was used to conduct all the analyses. A one-way analysis of variance (ANOVA) followed by Tukey's post hoc test was used to analyze differences among groups. A *p* value of <0.05 was considered statistically significant.

## 3. Results

### 3.1. BW and Epididymal Weight Changes after High-Fat Diet Fed for 8 Weeks

The initial BWs were similar among the groups. After 8 weeks' feeding, BWs of the high-fat diet groups were significantly higher than those of the NC group (HC 32.4 ± 1.5 g; HI 35.9 ± 0.8 g; HG 33.2 ± 0.9 g vs. NC 26.1 ± 1.7 g; *p* < 0.05). Differences in epididymal fat weights among groups had the same findings as BWs (HC 1.21 ± 0.05 g; HI 1.47 ± 0.61 g; HG 1.45 ± 0.35 g vs. NC 0.48 ± 0.02 g; *p* < 0.05).

### 3.2. Plasma Adipokine Levels after the High-Fat Diet Intervention

Diet-induced obesity resulted in upregulation of the plasma leptin concentration. However, no such changes were observed in adiponectin levels between the high-fat diet groups and the NC group (Table 3).

### 3.3. Blood EPC Population Changes after Limb Ischemia

**Figure 1:**
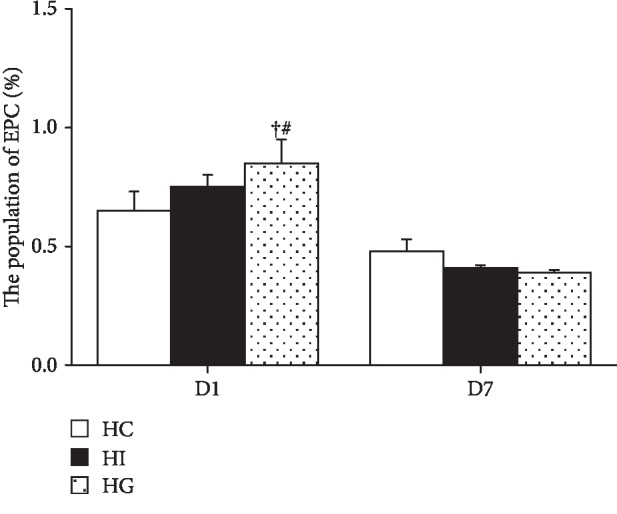
Percentage of blood total epithelial progenitor cells (EPCs). EPC populations were determined as the percentages of CD34^+^/CD133^+^/CD309^+^ cells among mononuclear cells. HC: high-fat sham-operated group; HI: high-fat ischemia group; HG: high-fat glutamine ischemia group. Values are presented as the means ± SEM. All data are representative of duplicate measurements on day 1 or 7 (*n* = 8 for the respective groups). Differences among groups were analyzed by a one-way ANOVA followed by Tukey's post hoc test. ^+^Significant differences from the HC group. ^#^Significant differences from the HI group at the same time point (*p* < 0.05).

No difference in EPC percentages was observed between the HC and HI groups (*p* > 0.05). The percentage of blood EPC in the HG group was significantly higher than that of the HC and HI groups on day 1; however, the increased EPC percentage returned to the level comparable to the HC group and exhibited no difference from the HI group by day 7 (Figure 1).

### 3.4. Plasma Leptin, Adiponectin, and EPC Mobilization-Related Protein Levels after Limb Ischemia

**Figure 2:**
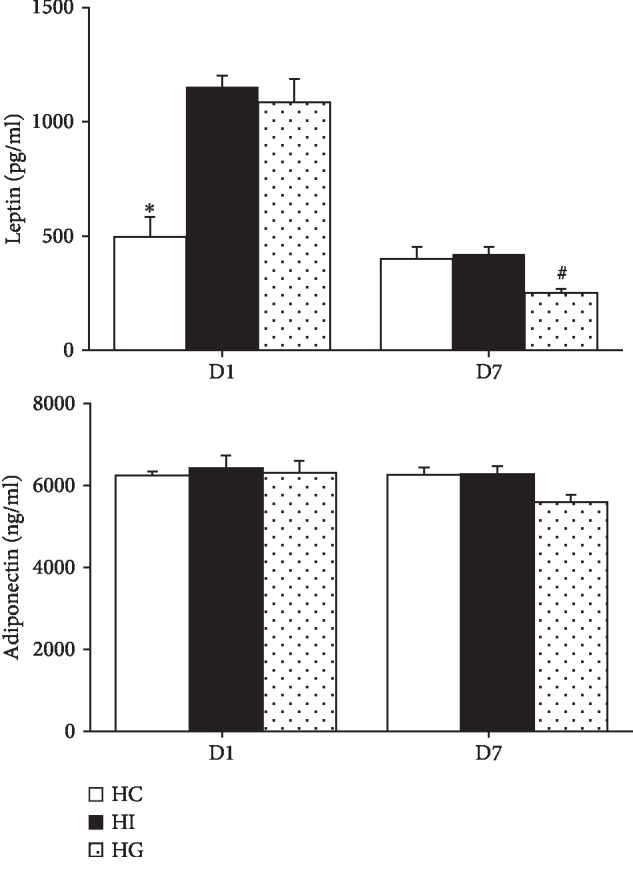
Plasma leptin and adiponectin concentrations in the sham-operated and ischemic groups. HC: high-fat sham-operated group; HI: high-fat ischemia group; HG: high-fat glutamine ischemia group. Values are expressed as the means ± SEM. All data are representative of duplicate measurements on day 1 or 7 (*n* = 8 for the respective groups). Differences among groups were analyzed by a one-way ANOVA followed by Tukey's post hoc test. ^∗^Significant differences from the two other groups. ^#^Significant differences from the HI group at the same time point (*p* < 0.05).

The HI and HG groups had higher leptin levels than the HC group on day 1. The elevated levels were downregulated to concentrations comparable to the HC group. The HG group had even lower plasma leptin levels than the HI group on day 7 after ischemia. There were no differences in adiponectin levels among the 3 groups on either day 1 or day 7 (Figure 2).

No significant differences in plasma stromal cell-derived factor (SDF) or vascular endothelial growth factor (VEGF) levels among the three groups were observed on either day 1 or 7. Matrix metalloproteinase- (MMP-) 9 levels were significantly higher in the HI and HG groups than those in the HC group on day 1. On day 7 after limb ischemia, the MMP-9 level was significantly higher in the HG group than that in the HC and HI groups (Table 4).

### 3.5. Antioxidant Capacity in Muscle Tissues after Limb Ischemia

**Figure 3:**
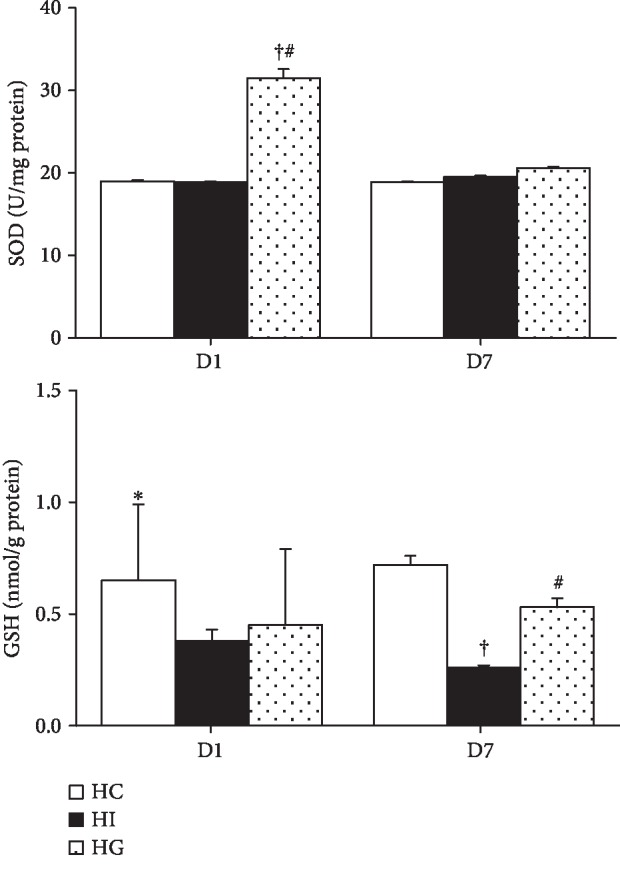
Superoxide dismutase (SOD) enzyme activity and reduced glutathione (GSH) content in ischemic muscle tissues. HC: high-fat sham-operated group; HI: high-fat ischemia group; HG: high-fat glutamine ischemia group. Values are expressed as the means ± SEM. All data are representative of duplicate measurements on day 1 or 7 (*n* = 8 for the respective groups). Differences among groups were analyzed by a one-way ANOVA followed by Tukey's post hoc test. ^∗^Significant differences from the two other groups. ^+^Significant differences from the HC group. ^#^Significant differences from the HI group at the same time point (*p* < 0.05).

The HG group had higher SOD activity than did the HC and HI groups on day 1. There were no differences in SOD activity among the HC, HI, and HG groups on day 7 after ischemia. Compared to the HC group, the ischemic groups (HI and HG) had lower GSH levels on day 1. However, on day 7, the HG group exhibited a higher GSH level than the HI group, but showed no difference from the HC group (Figure 3).

### 3.6. Gene Expression of Inflammatory and Anti-Inflammatory Mediator in Muscle Tissues after Limb Ischemia

**Figure 4:**
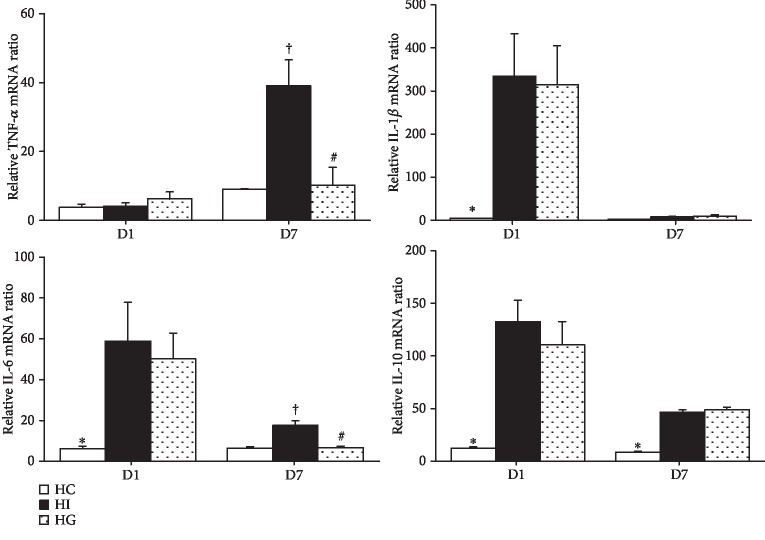
Expression of cytokine genes in muscle tissues. TNF-*α*: tumor necrosis factor-*α*; IL-1*β*: interleukin-1*β*; IL-6: interleukin-6; IL-10: interleukin-10; HC: high-fat sham-operated group; HI: high-fat ischemia group; HG: high-fat glutamine ischemia group. Values are expressed as the means ± SEM. All data are representative of duplicate measurements on day 1 or 7 (*n* = 8 for the respective groups). Differences among groups were analyzed by a one-way ANOVA followed by Tukey's post hoc test. ^∗^Significant differences from the two other groups. ^+^Significant differences from the HC group. ^#^Significant differences from the HI group at the same time point (*p* < 0.05).

Ischemic groups (HI and HG) had higher interleukin- (IL-) 1*β*, IL-6, and IL-10 mRNA expression levels than those in the HC group. There were no differences in tumor necrosis factor- (TNF-) *α* expression among the three groups on day 1 after ischemia. Compared to the HC group, the HI group had higher TNF-*α*, IL-6, and IL-10 expression levels on day 7. There were no differences in IL-1*β* and IL-10 mRNA expressions between the HI and HG groups. However, the HG group showed lower TNF-*α* and IL-6 expression levels on day 7 than the HI group (Figure 4).

### 3.7. Expressions of EPC Mobilization-Related Protein Genes in Muscle Tissue after Limb Ischemia

**Figure 5:**
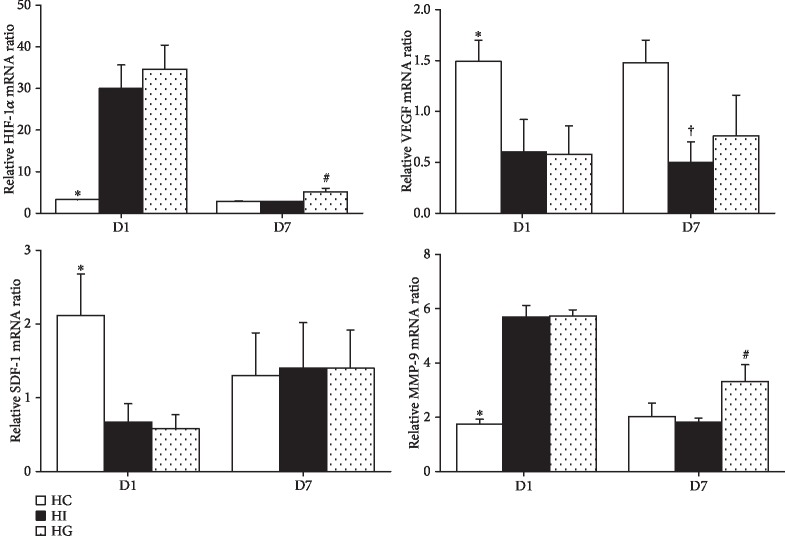
Messenger RNA expression of endothelial progenitor cell (EPC) mobilization-related protein in muscle tissues after limb ischemia. HIF-1*α*: hypoxia-inducible factor-1*α*; VEGF: vascular endothelial growth factor; SDF-1: stromal cell-derived factor; MMP-9: matrix metalloproteinase-9; HC: high-fat sham-operated group; HI: high-fat ischemia group; HG: high-fat glutamine ischemia group. Values are expressed as the means ± SEM. All data are representative of duplicate measurements on day 1 or 7 (*n* = 8 for the respective groups). Differences among groups were analyzed by a one-way ANOVA followed by Tukey's post hoc test. ^∗^Significant differences from the two other groups. ^+^Significant differences from the HC group. ^#^Significant differences from the HI group at the same time point (*p* < 0.05).

Compared to the HC group, the HI and HG groups showed higher hypoxia-inducible factor- (HIF-) 1 and MMP-9 mRNA levels but lower VEGF and SDF-1 mRNA expressions on day 1. Comparing between the HI and HG groups, the HG group showed upregulated HIF-1 and MMP-9 levels. There was no difference in SDF-1 expression between the two groups. The HI group had lower VEGF expression than the HC group, but this phenomenon was not observed between the HC and HG groups on day 7 (*p* < 0.05) (Figure 5).

## 4. Discussion

Obesity frequently results from excessive fat and energy intake. Fat accumulation and enlargement of adipocytes lead to macrophage infiltration into adipose tissues and create a state of chronic inflammation [26]. Underlain by oxidative stress, inflammation, and dyslipidemia that characterize obesity, hypercoagulability and hypofibrinolysis may occur which lead to a susceptibility to venous thromboses [27]. In this study, we used an animal model of obesity with limb ischemia to imitate a peripheral vascular thrombosis complicated by obesity. The main findings showed that obese mice treated with GLN increased the circulating EPC population shortly after ischemia and downregulated the expression of inflammatory chemokines in muscles that may have favorable effects in response to limb ischemia.

The central role of adipose tissues is lipid deposition. Additionally, they have major endocrine functions by secreting several hormones called “adipokines” that regulate lipid metabolism, vascular homeostasis, insulin sensitivity, immunity, etc. [28]. Expansion of fat depots as observed in obesity results in chronic inflammation and is linked to metabolic syndrome [29]. Leptin and adiponectin are notable adipokines secreted by adipose tissues. Leptin can be considered a hormone and a cytokine. As a hormone, it modulates energy homeostasis through thermoregulation, while as a cytokine, it promotes inflammatory responses that lead to low-grade inflammation in obesity [30]. Adiponectin is considered to have anti-inflammatory properties of inhibiting phagocytic activity and TNF-*α* production in macrophages [31]. In this study, 8 weeks' high-fat diet feeding in mice exhibited significantly increased BWs and epididymal weights. Although plasma adiponectin levels did not change after the weight gain, elevated leptin levels were noted in mice with high-fat diet-induced obesity. These findings indicate that excessive adipose tissue accumulation led to a proinflammatory response.

There are close links among obesity, inflammation, and thromboses [29]. Limb ischemia is a peripheral arterial occlusive disease with tissue inflammation that may occur in obese subjects complicated with vascular dysfunction. One of the important physiological functions of blood EPCs is to maintain the integrity of vascular tissues, which is especially important in vascular insults as occur during limb ischemia. Therefore, percentages of circulating EPCs and several EPC mobilization-associated protein expressions in plasma and muscle tissues were analyzed in this study. VEGF, an angiogenic factor, is responsible for the homing of EPCs [31]. HIF-1 is a regulatory factor of hypoxia-inducible gene transcription [32] and a chemokine, SDF-1, is responsible for EPC mobilization and recruitment [33]. MMP-9 degrades the extracellular matrix that is usually upregulated in tissue hypoxia [34] and promotes the release of EPCs from the bone marrow [35]. The results of our study showed increased plasma inflammatory cytokines and MMP-9 levels as well as upregulated muscle HIF-1*α* and MMP-9 expressions shortly after limb ischemia. These findings suggest that in response to inflammation and ischemia in the affected limb, a considerable extent of EPC mobilization may have occurred.

In this study, we found that obese mice pretreated with GLN resulted in several effects after limb ischemia that were not observed in groups without GLN. First, the GLN-treated group exhibited higher EPC percentages shortly after ischemia, which we think may have resulted from expressions of MMP-9 and HIF-1 in plasma and ischemic tissues that are responsible for recruiting EPCs [36, 37]. Our findings also showed that GLN administration led to higher HIF-1, MMP-9, and VEGF expressions at the late phase of ischemia in affected muscles. This may indicate more EPCs were recruited at the site of ischemic injury. However, the delayed elevation of EPC-associated protein expressions in the ischemic muscle was inconsistent with the rise of circulating EPCs at the early phase. Since EPCs in the blood may target the endothelial monolayer and integrate into it that ultimately become mature ECs during the repairing process to replace the damaged ECs [38], the percentage of circulating EPCs measured at late phase might not represent the actual extent of EPCs mobilized and integrated into the ischemic site. Second, obese mice with GLN supplementation resulted in lowered inflammatory mediator expressions including plasma leptin, muscle TNF-*α*, and IL-6 genes, especially at the late phase of ischemia. A study performed by Chen et al. investigated the effect of TNF-*α* on the activity of EPCs obtained from human peripheral blood. The authors found that incubation of EPCs with TNF-*α* reduced the proliferation, migration, and adhesive capacity of EPCs [39]. Decreased TNF-*α* and other inflammatory mediator expressions as exerted by GLN may be partly responsible for the enhancement of EPC mobilization that we observed. Third, high-fat diet supplemented with GLN increased muscle glutathione level and antioxidant enzyme activity after limb ischemia that may provide tissue protection against oxidative stress. GLN is the precursor of glutathione that is an important antioxidant in the body [40, 41]. In a streptozotocin-induced type 2 diabetes rodent model, the authors also found that feeding GLN-supplemented diet for 8 weeks decreased oxidative stress-related gene expressions and increased antioxidant capacity [21]. Obesity is a condition characterized by increased oxidative stress and chronic inflammation [42]. Obesity complicated with acute limb ischemia may further exacerbate the metabolic stress. There is a correlation between reactive oxygen species, inflammation, and EPC functions. Continuous process of oxidative stress will stimulate chronic inflammation that ultimately reduces the quantity and impair the quality of EPCs [43]. The antioxidative and anti-inflammatory properties of GLN may play important roles in promoting EPC mobilization in obesity with limb ischemia. However, vascular endothelial repair may involve complex molecular mechanisms, of which HIF-1- and VEGF-dependent pathways are part [44]. Although blood EPC percentage increased and higher EPC-mobilizing protein expression were observed in the GLN-supplemented group, reendothelialization and neovascularization in the ischemic muscles cannot be confirmed in this study. Determining the roles of GLN on regulating the mechanism that acts on EPCs mobilization and related vascular repair at the ischemic tissue needs further investigation.

## 5. Conclusions

The findings of this study revealed that obese mice pretreated with GLN-containing high-fat diet increased circulating EPC percentage, reduced muscle inflammatory mediator expression, and enhanced the antioxidant capacity at the site of the ischemic insult. To our knowledge, this is the first study to evaluate the effects of dietary GLN supplementation on changes of circulating EPCs in obese mice with acute limb ischemia. These findings provide basic information and imply that GLN use may have potential in attenuating oxidative stress and restoring damaged vascular endothelium in obesity accompanied with acute limb ischemia.

## Figures and Tables

**Table 1:** Compositions of the high-fat diets.

Ingredient	High fat (g/kg)	High fat+glutamine (g/kg)
Casein	259.13	194.36
Glutamine	—	54.55
L-cysteine	3.89	3.89
Corn starch	0.00	10.22
Maltodextrin	161.96	161.96
Sucrose	89.14	89.14
Cellulose	64.78	64.78
Soybean oil	32.39	32.39
Lard	317.44	317.44
Mineral mix^1^	12.96	12.96
Dicalcium phosphate	16.84	16.84
Calcium carbonate, 1H_2_O	7.13	7.13
Potassium citrate	21.38	21.38
Vitamin mix^2^	12.96	12.96
Total	1000	1000

^1^The compositions of the mineral mixture are listed as follows (mg/g): calcium phosphate dibasic, 500; sodium chloride, 74; potassium sulfate, 52; magnesium oxide, 24; potassium citrate monohydrate, 20; manganese carbonate, 3.5; ferric citrate, 6; chromium potassium sulfate, 0.55; zinc carbonate, 1.6; cupric carbonate, 0.3; potassium iodate, 0.01; sodium selenite, 0.01. ^2^The compositions of the vitamin mixture are listed as follows (mg/g): DL-*α*-tocopherol acetate, 20; nicotinic acid, 3; retinyl palmitate, 1.6; calcium pantothenate, 1.6; pyridoxine hydrochloride, 0.7; thiamin hydrochloride, 0.6; riboflavin, 0.6; cholecalciferol, 0.25; D-biotin, 0.05; menaquinone, 0.005; and cyanocobalamin, 0.001.

**Table 2:** Sequences of oligonucleotide primers used in the PCR amplification.

	Primer sequences (5′ → 3′)	
GAPDH	Forward	TGCACCACCAACTGCTTAG
	Reverse	GGATGCAGGGATGATGTTC

HIF-1*α*	Forward	CGCCTCTGGACTTGTCTCTT
	Reverse	TTCTTCTCGTTCTCGCCGC

VEGF	Forward	GATCATGCGGATCAAACCTC
	Reverse	AATGCTTTCTCCGCTCTGAA

SDF-1	Forward	CAGCCGTGCAACAATCTGAAG
	Reverse	CTGCATCAGTGACGGTAAACC

MMP-9	Forward	CCAGCCGACTTTTGTGGTCT
	Reverse	TGGCCTTTAGTGTCTGGCTG

TNF-*α*	Forward	AAATGGGCTCCCTCTCATCAGTTC
	Reverse	TCTGCTTGGTGGTTTGCTACGAC

IL-1*β*	Forward	TGCCACCTTTTGACAGTGATG
	Reverse	ATGTGCTGCTGCGAGATTT

IL-6	Forward	TCCTACCCCAACTTCCAATGCTC
	Reverse	TTGGATGGTCTTGGTCCTTAGCC

IL-10	Forward	ACCTGGTAGAAGTGATGCCCCAGGCA
	Reverse	CTATGCAGTTGATGAAGATGTCAAA

GAPDH: glyceraldehyde 3-phosphate dehydrogenase; HIF: hypoxia-inducible factor; VEGF: vascular endothelial growth factor; SDF: stromal cell-derived factor; MMP: matrix metalloproteinase; TNF: tumor necrosis factor; IL: interleukin.

**Table 3:** Plasma leptin and adiponectin concentrations in the normal and high-fat diet groups.

	Leptin (pg/ml)	Adiponectin
NC	46.21 ± 6.5	5.87 ± 0.32
HC	488.2 ± 52.22^∗^	6.21 ± 0.13
HI	558.32 ± 32.51^∗^	6.31 ± 1.31
HG	502.25 ± 45.51^∗^	6.15 ± 1.85

Data are expressed as the means ± SEM. NC: normal control group; HC: high-fat control group; HI: high-fat ischemia group; HG: high-fat glutamine ischemia group. All data are representative of duplicate measurements (*n* = 8). Differences among groups were analyzed by an unpaired *t*-test. ^∗^Significant differences from the NC group.

**Table 4:** Plasma endothelial progenitor cell (EPC) mobilization-related protein levels after limb ischemia.

	HC	HI	HG
SDF-1 (pg/ml)			
D1	1.41 ± 0.23	0.99 ± 0.11	0.78 ± 0.17
D7	1.77 ± 0.35	1.12 ± 0.21	0.84 ± 0.26
VEGF (pg/ml)			
D1	57.37 ± 4.37	55.64 ± 2.77	54.64 ± 3.29
D7	36.06 ± 4.61	35.62 ± 3.02	31.75 ± 2.44
MMP-9 (ng/ml)			
D1	37.50 ± 2.24^∗^	53.61 ± 4.77	49.66 ± 2.41
D7	40.31 ± 1.61	43.62 ± 3.22	58.68 ± 5.59^∗^

Data are expressed as the means ± SEM. NC: normal control group; HC: high-fat control group; HI: high-fat ischemia group; HG: high-fat glutamine ischemia group; SDF: stromal cell-derived factor; VEGF: vascular endothelial growth factor; MMP: matrix metalloproteinase. All data are representative of duplicate measurements on day 1 or 7 (*n* = 8 for the respective groups). Differences among groups were analyzed by a one-way ANOVA followed by Tukey's post hoc test. ^∗^Significant differences from the two other groups.

## Data Availability

No data were used to support this study.
